# Clinical characterizations of three adults with genetically confirmed spinal muscular atrophy: a case series

**DOI:** 10.1186/s13256-022-03633-y

**Published:** 2022-11-15

**Authors:** Cempaka Thursina Srie Setyaningrum, Indra Sari Kusuma Harahap, Dian Kesumapramudya Nurputra, Mawaddah Ar Rochmah, Ahmad Hamim Sadewa, Giga Hasabi Alkarani, Nur Imma Fatimah Harahap

**Affiliations:** 1 Department of Neurology, Faculty of Medicine, Public Health and Nursing, Universitas Gadjah Mada, Yogyakarta, Indonesia; 2 Department of Pediatrics, Faculty of Medicine, Public Health and Nursing, Universitas Gadjah Mada, Yogyakarta, Indonesia; 3 Department of Biochemistry, Faculty of Medicine, Public Health and Nursing, Universitas Gadjah Mada, Yogyakarta, Indonesia; 4 Department of Clinical Pathology, Faculty of Medicine, Public Health and Nursing, Universitas Gadjah Mada, Yogyakarta, Indonesia

**Keywords:** Spinal muscular atrophy, Clinical characterization, Diagnosis, Indonesia

## Abstract

**Background:**

Spinal muscular atrophy is a recessively inherited autosomal neuromuscular disorder, with characteristic progressive muscle weakness. Most spinal muscular atrophy cases clinically manifest during infancy or childhood, although it may first manifest in adulthood. Although spinal muscular atrophy has come to the era of newborn screening and promising treatments, genetically confirmed spinal muscular atrophy patients are still rare in third world countries, including Indonesia.

**Case presentations:**

We presented three Indonesian patients with spinal muscular atrophy genetically confirmed during adulthood. The first case was a 40-year-old male who presented with weakness in his lower limbs that started when he was 9 years old. At the age of 16 years, he could no longer walk and started using a wheelchair. He first came to our clinic at the age of 38 years, and was diagnosed with spinal muscular atrophy 2 years later. The second patient was a 58-year-old male who presented with lower limb weakness since he was 12 years old. Owing to the geographical distance and financial problems, he was referred to our clinic at the age of 56 years, when he already used a walker to walk. Lastly, the third patient was a 28-year-old woman, who was in the first semester of her second pregnancy, and who presented with slowly progressing lower limb weakness. Her limb weakness began at the age of 8 years, and slowly progressed until she became dependent on her wheelchair 8 years later until now. She had successfully given birth to a healthy daughter 3 years before her first visit to our clinic. All three patients were diagnosed with neuromuscular disorder diseases, with the differential diagnoses of Duchenne muscular dystrophy, spinal muscular atrophy, and Becker muscular dystrophy. These patients were finally confirmed to have spinal muscular atrophy due to *SMN1* deletion by polymerase chain reaction and restriction fragment length polymorphism.

**Conclusions:**

Many genetic diseases are often neglected in developing countries owing to the difficulty in diagnosis and unavailable treatment. Our case series focused on the disease courses, diagnosis difficulties, and clinical presentations of three patients that finally lead to diagnoses of spinal muscular atrophy.

## Introduction

Spinal muscular atrophy (SMA) is a neuromuscular disorder characterized by degenerations of anterior horn cells in the human spinal cord and subsequent loss of function in motor neurons leading into progressive muscular atrophy, muscle weakness, and muscle paralysis [1–3]. The classifications of SMA are based on the age of onset and maximum motor function achieved. The first is SMA type I, with onset in the first month of life in which the patient is unable to sit/move unsupported and also needs breathing support, with a chance of survival only up to 2 years old. The second is SMA type II, with onset between 6 and 18 months old and with motor ability to sit alone without support. The third is SMA type III, with onset between 18 months–30 years old. The patients can stand and walk without support. The fourth is SMA type IV, which is the mildest type of SMA with onset in adulthood, in which the patient tends to have normal mobility with mild muscle weakness [4, 5].

The survival motor neuron (SMN) genes, *SMN1* and *SMN2*, have been identified as the genes responsible for SMA. They exist in chromosome 5q13.2 as highly homologous gene copies. *SMN1* is now considered as the primary gene responsible for SMA, because *SMN1* is completely deleted in more than 95% of SMA patients and is deleteriously mutated (intragenic mutations) in the rest of patients [6]. *SMN2* has been considered as a dispensable gene because absence of *SMN2* is frequently found in control individuals, but it is now considered to be a modifying factor of the SMA phenotype, which is supported by the fact that a high copy number of *SMN2*may be related to the milder phenotype of SMA [7, 8]. According to recent research, the incidence of SMA cases owing to mutation of the *SMN1* gene is approximately 1 in 10,000 newborns, with a prevalence of approximately 1–2 per 100,000 people [5].

In Indonesia, genetic neuromuscular diseases, including SMA, are commonly misdiagnosed owing to the similar clinical presentations with other neuromuscular disorders, such as Duchenne muscular dystrophy, Becker muscular dystrophy, multiple sclerosis, amyotropic lateral sclerosis, and even poliomyelitis. To establish the diagnosis of SMA, molecular genetic analysis is necessary to find the deletion or intragenic mutation in *SMN1* [6]. Therefore, genetic diagnosis centers should be available to perform these genetic analyses.

Moreover, supporting examinations are not always available to further investigate the patients in local healthcare facilities, particularly in remote areas. To date, genetic diagnosis centers for SMA in Indonesia have been established mostly in Java Island. However, it is difficult to send patients from different islands of Indonesia for genetic analysis owing to the patients’ limited mobility, geographical landmarks, and financial problems. However, current technologies of genetic testing are rapidly developing, and there are many new methods that are much simpler, easier, and more efficient for examining genetic diseases [9].

**Fig. 1:**
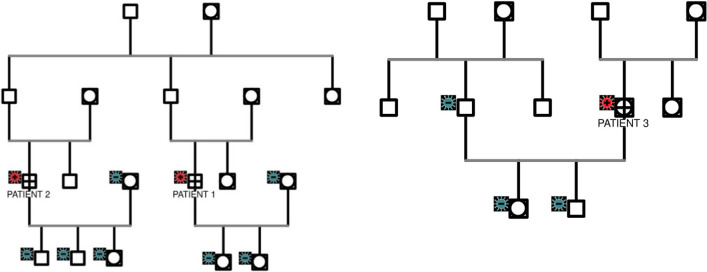
Family genogram. From this, we identified that patient 1 and patient 2 were cousins. Neither of their wives nor any children have any deletion on the *SMN1* gene. No further information was available about other family members because they did not undergo the SMA genetic testing

In our case series, we report three patients with neuromuscular complaints who were found to have *SMN1* deletions, thus confirming the diagnosis of SMA. All of them were Javanese and lived in remote rural areas in Indonesia. Patients 1 and 2 were related as cousins. Besides these two patients, there were no other family members who suffered from the same disease (Fig. 1). These case series focused on the disease courses, diagnosis difficulties, and clinical presentations of the three patients that lead to the SMA diagnosis.

## Case presentations

### Patient 1

A 40-year-old Indonesian male in a wheelchair presented with weakness in his lower limbs. His lower limb weakness started when he was 9 years old, when he used to complain of being easily fatigued and weakness in his legs after playing with his friends. In his recall during history taking, he denied frequently using his upper limbs to push his legs upright after squatting or sitting, having breathing trouble, feeling pain or muscle cramps in his legs, frequently stumbling or losing handgrip, or having breast enlargement. At the time, he was still able to walk to his school, although he often needed to stop owing to the weakness in his legs. By the age 16 years, he could no longer walk and he started using a wheelchair. Not until the age of 18 years, owing to the lack of family knowledge and financial support, did his family seek medical advice at a district hospital. He underwent physiotherapy but he did not go to the central hospital until the age of 38 years, when he first came to our clinic.

On clinical examination, we found weakness of the four limbs, with slightly decreased strength in the upper limbs and markedly decreased strength in the lower limbs. The weakness was flaccid, with decreased muscle tones and hyporeflexia, particularly in his lower limbs. Gower sign was negative and extensor plantar response was not found. Genetic testing showed a deletion of exon 7 in the *SMN1* gene, confirming the diagnosis of SMA. We planned a routine medical rehabilitation for the patient, but he could only come for several meetings owing to his family’s financial problems.

The patient was a tailor and was able to perform his daily activities using his upper limbs, even performing fine motor activities such as knitting. The patient lived with his wife and his two children, who had normal genetic testing. The follow-up of this patient was performed monthly in the outpatient clinic until 1 year after his diagnosis of SMA. There were no major complaints, such as breathing difficulty, until his last visit. He decided to stop coming to our clinic owing to geographical and financial problems.

### Patient 2

A 58-year-old Indonesian male presented with lower limb weakness and used a walker to help him walk. This patient was the cousin of Patient 1. He came to our clinic on the suggestion of Patient 1 after he was diagnosed with SMA. His lower limb weakness started when he was 12 years old, when he frequently complained of weakness of his lower limbs after playing with friends or walking to school. Although he had weakness in his lower limbs, he was still able to finish school and work as a factory worker until the age of 25 years. In his recall during history taking, he denied frequently using his upper limbs to push his legs upright after squatting or sitting, having breathing trouble, feeling pain or muscle cramps in his legs, frequently stumbling or losing handgrip, or having breast enlargement.

During the physical and neurological examinations, we found weakness in all four limbs, but the patient was able to stand up by himself and walk a few steps without assistance or his walker. There was a slight decrease in muscle strength in his upper limbs but a marked decreased in his lower limbs. The weakness was flaccid, with a slight decrease in muscle tone, especially in the lower limbs. Gower sign was negative, and extensor plantar response was negative. Genetic testing showed a deletion of exon 7 *SMN1* gene and confirmed the diagnosis of SMA. Medical rehabilitation including physiotherapy and occupational therapy were planned, but he could only attend two meetings owing to his financial problems and the difficult trip to the hospital.

This patient was a factory worker and still able to walk and perform his job by himself. The follow-up of this patient was performed up to 2 months after the diagnosis of SMA was established. He decided to decline the medical rehabilitation programs owing to financial and geographical problems.

### Patient 3

A 28-year-old Indonesian woman who was pregnant with her second child, came with complaints of pain in her buttocks that radiated to her stomach and chest. The patient was unable to walk and dependent on her wheelchair. The patient said that she had had this condition since she was a child. In her recall during history taking, the patient was able to walk at 2 years old but she started to have difficulty in standing and walking at the age of 8 years old. She was taken to see a doctor and was told that she might have a rare neuromuscular disease, with one differential diagnosis being SMA. She was scheduled for medical rehabilitation, including physiotherapy and occupational therapy, but she could only attend the program for 1 year. She did not continue the program owing to financial problems. At the age of 16 years, she started using a wheelchair for her activity but was still able to walk by herself for short distances. The patient had her first child at 25 years of age. After giving birth, she was no longer able to stand or walk without assistance. She started to be dependent to her wheelchair all day. She was in her first trimester of her second pregnancy at the time of presentation, and was referred to our clinic for better examination and genetic testing for SMA.

During the physical and neurological examinations, the patient was unable to stand or move her legs. Her spine was observed to have scoliosis. There was weakness in all four limbs, especially the lower limbs. The weakness was flaccid and there was a decrease in the muscle tone of the upper limbs and a major decrease in the lower limbs. In the motoric strength examination, there was a massive decrease in the motor strength of her lower limbs. Extensor plantar response was not found, and Gower sign was negative. The genetic results showed a deletion of *SMN1* exons 7 and exon 8, confirming the diagnosis of SMA.

In her daily life, the patient worked helping in her aunt’s local food business by performing tasks that she could do while sitting on her wheelchair. When this report was made, the patient had successfully delivered her second child. The follow-up of this patient was routinely performed once a month from the first trimester of her second pregnancy until 3 months after she delivered her second child.

## Discussion and conclusions

All of the patients in the presented cases were diagnosed with SMA type III because their onsets were between 18 months and 30 years old. Patients with SMA type 3 usually develop more problems with back pain and can include disorders of the backbone such as scoliosis [10–12]. Before we could reach the diagnosis of SMA type III, some considerations of other neuromuscular disorders were also taken into account. Although all the patients presented during adulthood, the disease onset started when they were children. Therefore, genetic neuromuscular disorders were suspected. Upon history taking and physical examinations, we eliminated Duchenne muscular dystrophy owing to the absence of Gower signs and the cardiomyopathy signs, as well as shortness of breath [13]. Kennedy disease often shows gynecomastia and decreased fertility, but these patients were not infertile and did not have gynecomastia. Another possible differential diagnosis was amyotrophic lateral sclerosis. Amyotrophic lateral sclerosis presents with motor symptoms such as extensor plantar response, increase muscle tone, lower limbs pain, and muscle cramps that occur as the result of degeneration of both upper and lower motor neurons [14]. However, the three patients did not show any extensor plantar response and muscle cramps. The third patient is female and her clinical manifestations may also fit Becker muscular dystrophy (BMD). The patient had no history of speech disturbance, shortness of breath, or toe walking, which may support the diagnosis of BMD. BMD is a rare disease almost exclusively in males, female carriers may only present with cardiomyopathy. Sometimes carrier females may have mild muscle weakness with approximately 22% of carriers become symptomatic [15]. Owing to the limitation of genetic or molecular testing in our medical or research facilities, it is difficult to reach a definitive diagnosis of a genetic disease. In addition, if the testing is available, the cost is expensive and not covered by the national health care insurance. Clinicians should pay attention in the clinical details in history taking and physical examinations to narrow down the differential diagnoses and to plan further diagnostic testing. In our cases, effort was required to convince the patients to perform the genetic examinations despite the financial, geographical, and availability problems to establish a diagnosis.

As in other forms of SMA, muscle fatigue, weakness, and atrophy start in lower limbs, then there is proximal predominance, and spreading into the upper limbs and trunk in an ascending manner [4, 12]. When children start to walk, run, or even climb stairs they usually fall and show slow disease progression. Falls are increasingly frequent as their body changes during puberty when they will typically gain more weight. As they get older, the symptoms also get more progressive, with muscle weakness and complaints of fatigue as the most common [12]. From this brief explanation, it can be seen that all of the patients reported here had a similar progression of SMA type 3. Muscle weakness and inability to stand and walk independently are the most progressive symptoms that appeared. Some patients with SMA may also complain about general pain that mostly is from the back. Scoliosis occurs in almost all non-ambulant individuals with SMA. When untreated, scoliosis can cause chest deformities with subsequent respiratory restriction [1]. In this report, one of the patients had severe scoliosis but it did not affect her respiratory function. SMA type 3 usually has a slow progression. However, deformities including those in the vertebral column and lower limbs are frequent, and may lead to wheelchair use [16]. With this variety of clinical symptoms and presentations of SMA type 3, it may helpful that the patient knows about their condition as soon as they can. An early diagnosis may be helpful to involve a neurologist for confirming the diagnosis of SMA, but this genetic testing technology is still not available in every country, especially in developing countries such as Indonesia, where it is only available in only a few central hospitals. This may prevent early detection and cause a delay in diagnosing any genetic disorders, including SMA.

**Fig. 2:**
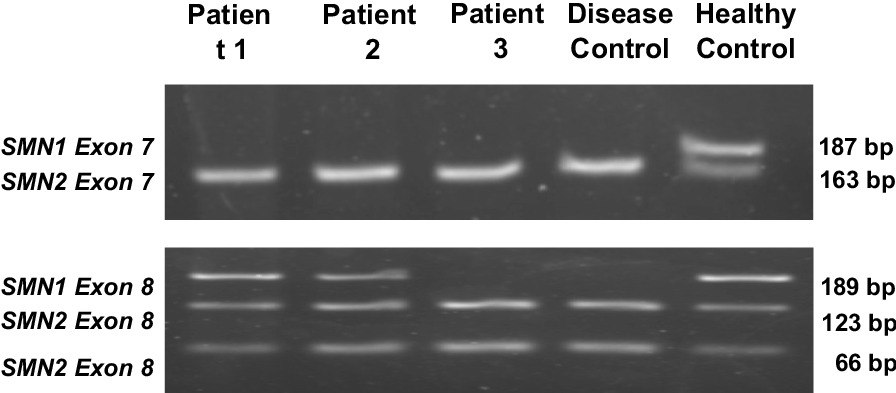
The genetic test of *SMN1* and *SMN2* from patients. From this figure we determined that all the patients had a deletion on *SMN1* exon 7 gene. In addition, Patient 3 showed a deletion of *SMN1* exon 8

The confirmed SMA diagnosis of the three patients, shown by genetic testing, was identified from the deletion of *SMN1* exon 7, and in addition, the third patient also had deletion in *SMN1* exon 8 (Fig. 2). An isolated deletion in *SMN1* exon 7 usually occurs in patients with SMA type II and type III, and deletion in *SMN1* exon 8 gene is also associated with SMA [17]. Some authors argue that exon 8 is untranslated, therefore, an *SMN* gene containing such a mild mutation could be fully functional [18]. Accordingly, it has been argued that the untranslated sequences of genes may have regulatory function at the transcriptional level, thus, mutations on this region may impair these biological functions. One report found that two SMA patients with deletion of *SMN1* exon 8 gene, but who retained the exon 7 gene had no significant clinical features [17]. Of interest, genotype/phenotype correlations in SMA found that the type of genes deleted markedly affected the severity of the disease [18, 19].

To establish the diagnosis of SMA, it is necessary to find the deletion or intragenic mutation in *SMN1*. Various polymerase chain reaction (PCR) technologies have been developed to separate *SMN1* and *SMN2* amplification, for example, PCR and single-strand conformation polymorphism (PCR–SSCP), PCR and restriction fragment length polymorphism (PCR–RFLP), radio-isotope competitive PCR and RFLP, PCR and denaturing high-performance liquid chromatography (DHPLC), real-time PCR with gene-specific primers, multiplex ligation probe amplification (MLPA), tetra-primer PCR, high-resolution melting analysis, and modified competitive oligonucleotide primers PCR [9, 20]. In Indonesia, SMA and other genetic neuromuscular disorders often become the last differential diagnosis to be considered owing to their rarity, the similarity of the clinical presentations with other neuromuscular disorders, and the difficulty in further investigating by molecular genetic analysis. None of the aforementioned PCR technologies are readily available and easily accessible for general practitioners and patients in Indonesia. In addition, the lack of national health insurance for genetic testing, the shortage of standardized operational procedures for genetic molecular testing, and the inadequacy of community knowledge and awareness of genetic diseases, in addition to the scarcity of the genetic experts for genetic consultation make the situation more difficult in Indonesia to establish genetic disease diagnoses, including SMA. Through publishing this case series, we also want to inform and describe three SMA cases that can hopefully lead more scientists, researchers, and doctors to be more aware and pay more attention to these problems of genetic diseases and the difficulty in their diagnoses, especially in developing countries such as Indonesia. Many genetic diseases are often neglected in developing countries owing to the difficulty in diagnosis and unavailable treatments. However, the new era for the screening, diagnosis, and treatment of SMA has come. This new science should be introduced to every clinician, researcher, and policy maker who deals with genetic diseases, such as spinal muscular atrophy.

## Data Availability

Any statement, information, and data in this case series was already obtained an approval and acceptance from the subjects and their family.

## Abbreviations

SMA: Spinal muscular atrophy
SMN-1: Survival motor neuron-1
SMN-2: Survival motor neuron-2
BMD: Becker muscular dystrophy
DMD: Duchenne muscular dystrophy
PCR: Polymerase chain reaction
PCR-SSCP: Polymerase chain reaction and single-strand conformation polymorphism
PCR-RLFP: Polymerase chain reaction–restriction fragment length polymorphism
DHPLC: Denaturing high-performance liquid chromatography
MLPA: Multiplex ligation probe amplification
